# Agrimonia: a dataset on livestock, meteorology and air quality in the Lombardy region, Italy

**DOI:** 10.1038/s41597-023-02034-0

**Published:** 2023-03-18

**Authors:** Alessandro Fassò, Jacopo Rodeschini, Alessandro Fusta Moro, Qendrim Shaboviq, Paolo Maranzano, Michela Cameletti, Francesco Finazzi, Natalia Golini, Rosaria Ignaccolo, Philipp Otto

**Affiliations:** 1 University of Bergamo, Dept. of Economics, Via dei Caniana 2, 24127 Bergamo, Italy; 2 University of Torino, Dept. of Economics and Statistics, Lungo Dora Siena 100A, 10153 Torino, Italy; 3 Leibniz University Hannover, Institute of Cartography and Geoinformatics, Appelstrasse 9a, 30167 Hannover, Germany; 4 University of Milano-Bicocca, Dept. of Economics, Management and Statistics, Piazza dell’Ateneo Nuovo 1, 20126 Milano, Italy; 5 Fondazione Eni Enrico Mattei (FEEM), Corso Magenta 63, 20123 Milano, Italy

**Keywords:** Environmental impact, Agriculture

## Abstract

The air in the Lombardy region, Italy, is one of the most polluted in Europe because of limited air circulation and high emission levels. There is a large scientific consensus that the agricultural sector has a significant impact on air quality. To support studies quantifying the role of the agricultural and livestock sectors on the Lombardy air quality, this paper presents a harmonised dataset containing daily values of air quality, weather, emissions, livestock, and land and soil use in the years 2016–2021, for the Lombardy region. The daily scale is obtained by averaging hourly data and interpolating other variables. In fact, the pollutant data come from the European Environmental Agency and the Lombardy Regional Environment Protection Agency, weather and emissions data from the European Copernicus programme, livestock data from the Italian zootechnical registry, and land and soil use data from the CORINE Land Cover project. The resulting dataset is designed to be used as is by those using air quality data for research.

## Background & Summary

**Fig. 1:**
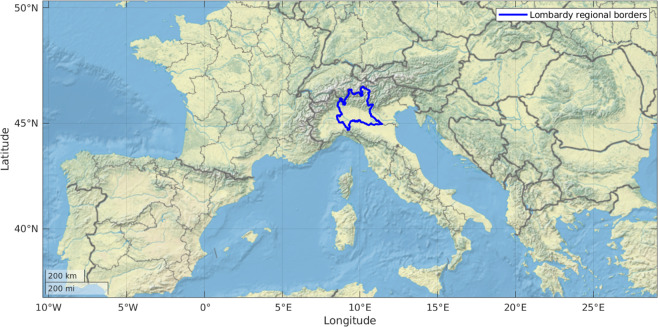
Lombardy region (blue line) in Northern Italy, surrounded by the Alps.

Air pollutants may be categorised as primary or secondary. Primary pollutants are directly emitted to the atmosphere, whereas secondary pollutants are formed in the atmosphere from precursor gases through chemical reactions and microphysical processes. One of the key precursor gases for secondary particulate matter (PM) is ammonia (NH_3_). This holds true for both large PM with aerodynamic diameter less then 10 *μm* (PM_10_) and fine PM with aerodynamic diameter less then 2.5 *μm* (PM_2.5_). There is a large scientific consensus that livestock and fertilisers are responsible for ammonia emissions^1,2^. In Europe, around 90% of ammonia emissions originate from the agricultural sector^3^, while, in the Italian Lombardy region, shown in Fig. 1, up to 97% of ammonia emissions are linked to the agricultural sector^4^. According to Lombardy Environment Protection Agency^5^, ammonia reacts with nitric acid and that reaction product can contribute up to 60% of the PM_10_ mass concentration.

This paper presents an open-access spatiotemporal dataset, named the Agrimonia dataset^6^, which includes several environmental variables that have been harmonised at the same spatial and temporal resolution. The dataset has been developed within the AgrImOnIA project framework (*Agriculture Impact On Italian Air* - https://agrimonia.net) which aims at assessing the role of the livestock sector on the air quality in the Lombardy region, and at allowing comparisons with other European regions. In particular, the AgrImOnIA project aims at providing spatially resolved geostatistical maps able to describe the local impact of livestock and its variations due to mitigation policies. The Agrimonia dataset provides the user with emissions data, including ammonia, agricultural information and air pollution in a common table. In general, handling spatiotemporal data from several sources is a challenge faced in various research fields and represents an interdisciplinary topic. Along these lines, the Agrimonia dataset aims to be a standard for European intercomparisons and uses data available at the European level rather than local ones, as much as possible.

The Agrimonia dataset could be useful to other researchers, for example, in comparing urban air pollution and rural air quality^7,8^. Other uses of the dataset may move toward the study of different livestock management techniques and organic products^9^ or for epidemiological studies, which aim to assess the impact of agricultural emissions on the mortality attributable to air pollution^10^. Additionally, the land use and land cover variables included in the Agrimonia dataset, provide indices of the urbanisation degree^11^, ecosystems conservation^12^, and natural resources exploitation, allowing for assessing the degree of local sustainable development^13^.

The rest of this paper is organised as follows: in the Methods Section, we describe the data sources and the transformations applied to harmonise the dataset, as well as the methodologies used to impute missing data and handle negative values. In the Data records Section, we describe our dataset and the various associated metadata files. Finally, the quality of the dataset and the method adopted are discussed in the Technical validation Section.

## Methods

The Agrimonia dataset includes satellite data, model output and *in situ* measurements with different spatial and temporal resolutions from national and international agencies. Therefore, to combine the different datasets, a processing step is necessary. The remainder of this section describes the data sources and the harmonisation process applied to the different input data to make them homogeneous in time, with a daily resolution, and space, at the air quality station level.

### Source data description

**Fig. 2:**
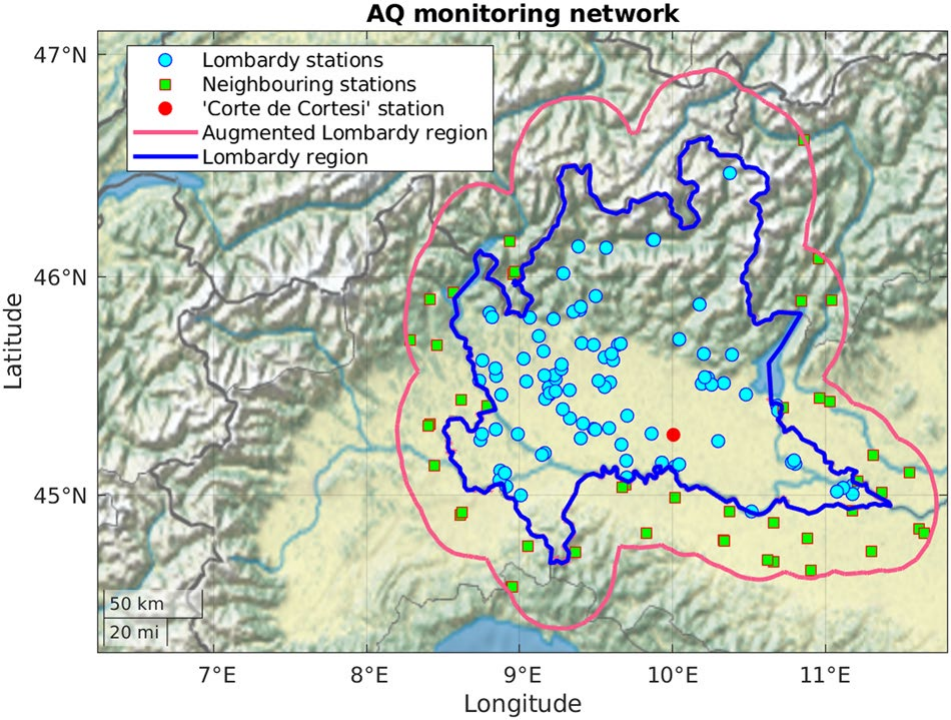
AQ network of *S* = 141 stations in the augmented Lombardy region (pink boundaries): *S*_1_ = 93 stations are inside the Lombardy region (blue boundaries) and *S*_2_ = 48 in the 0.3° buffer defining the neighbouring area between blue and pink boundaries. Station named ‘Corte de Cortesi’ is marked as a red circle and used as a reference throughout the paper.

The data presented are related to five dimensions: air quality (AQ), weather and climate (WE), pollutants’ emissions (EM), livestock (LI) and land and soil characteristics (LA). Because geostatistical methods can use neighbouring territory information^14^ for improving the overall predictive capability close to the borders, we take into account an area around Lombardy region by applying a 0.3° buffer over the regional borders as shown in Fig. 2. The neighbouring area intersects several regions. The various data sources used to create the Agrimonia dataset are summarised in Table 1 and described in the following subsections that detail spatiotemporal resolution and availability.

**Table 1:** Sources of the Agrimonia dataset.

Dimension	Source	Data description	Spatial coverage	Temporal resolution
Air quality (AQ)	EEA - Air pollution section	Air pollutants concentrations^39^	Europe	Daily/Hourly/Bi-hourly
	ARPA Lombardy - Air quality section	Air pollutants concentrations^16^	Lombardy	Daily/Hourly/Bi-hourly
Weather (WE)	Copernicus Climate Change Service (ERA5)	Estimates of atmospheric and land cover variables^17,18^	Europe	Hourly
Emission (EM)	Copernicus Atmosphere Monitoring Service (CAMS)	Emission inventories^20^	Global	Monthly
Livestock (LI)	National Data Bank (BDN) of the Zootechnical Registry	Livestock inventories^23^	Italy	Biannual
Land cover (LA)	Copernicus Land Monitoring Service (CLMS)	Corine land use classification^25^	Europe	Only 2018
	Lombardy Region Agriculture Information System (SIARL)	Cultivation type classification^27^	Lombardy	Annual
	Copernicus Climate Change Service (ERA5)	Estimates of land cover variables^18^	Europe	Hourly

#### Air quality

The AQ data are pollutant concentrations [*μg*/*m*^3^] sampled at *S* = 141 ground-level monitoring stations, irregularly located over the augmented Lombardy region, as shown in Fig. 2. For the Lombardy area, the AQ data of 93 stations are retrieved by the environment protection agency of the Lombardy region (ARPA Lombardy, hereinafter ARPA), while outside of Lombardy, data are obtained by European Environment Agency (EEA) from another 48 stations. Inside Lombardy, the variables listed in Table 2, namely PM2.5, PM10, NO2, NO_*x*_, CO, SO2 and NH3 with a daily temporal resolution come from the open data system of ARPA and are validated under EEA protocols. Instead, hourly data about NH3 for the station called ‘Bergamo Via Meucci’ come from experimental monitoring campaigns implemented by ARPA according to laboratory best practices^5,15^, but are not formally validated under the same EEA protocols. For the neighbouring EEA stations, data are either daily or hourly and cover all the above variables but NH_3_.

**Table 2:** AQ pollutants concentrations data sources, descriptions, sampling frequency and number of sensors (or stations) per pollutant, which gives a total of 513 sensors throughout extended Lombardy.

Pollutant	Description	Source	Temporal resolution	Number of sensors
PM_10_	Particulate matter with an aerodynamic diameter of less than 10 µm	ARPA Lombardy/EEA	Daily/hourly	107
PM_2.5_	Particulate matter with an aerodynamic diameter of less than 2.5 µm	ARPA Lombardy/EEA	Daily/hourly/bi-hourly	54
CO	Carbon monoxide	ARPA Lombardy/EEA	Hourly	56
NH_3_	Ammonia	ARPA Lombardy	Daily/hourly	10
NO_*x*_	Nitrogen oxides	ARPA Lombardy/EEA	Hourly	110
NO_2_	Nitrogen dioxide	ARPA Lombardy/EEA	Hourly	136
SO_2_	Sulphur dioxide	ARPA Lombardy/EEA	Hourly/bi-hourly	40

In this work, data from the open data system of ARPA (https://www.arpalombardia.it/Pages/Aria/qualita-aria.aspx, accessed on 5 February 2022) are collected using the ARPALData package written in R language and available on CRAN (version 1.2.3) (https://cran.r-project.org/web/packages/ARPALData/index.html, accessed on 5 February 2022). The data used for the Lombardy neighbouring areas come from the open access service of the EEA (https://www.eea.europa.eu/themes/air, accessed on 5 February 2022). To get an overview of AQ data used, Table 2 summarises the pollutants selected, their sources and the number of sensors available for each pollutant.

At each AQ station, concentration data of possibly different subsets of pollutants are gathered. For each AQ station, pollutants, spatial location, altitude, station type and other information are available in the station registry metadata file named ‘Metadata_monitoring_network_registry_v_2_0_1.csv’ provided with the Agrimonia dataset^6^. EEA and ARPA classify air quality stations according to the land use and emission context^16^ as summarised in Table 3.

**Table 3:** Station type taxonomy of EEA/ARPA, initials used in metadata files, and distribution of the 131 stations in extended Lombardy. The land use is classified as U, S, R while the emission context is classified as B, T, I. This classification is not available to the authors at the time of writing for the remaining ten stations of the 141-stations AQ network.

		Background	Traffic	Industrial
		B	T	I
Urban	U	42	36	3
Suburban	S	25	1	4
Rural	R	18	—	2

#### Weather

Meteorological data are obtained from the Copernicus Climate Change Service (https://climate.copernicus.eu/, accessed on 27 April 2022) through the ERA5 datasets containing the numerical model output computed by the European Centre for Medium-Range Weather Forecasts (ECMWF). ERA5 is the fifth generation ECMWF reanalysis of the global climate for the past decades. The reanalysis combines model data with observations from across the world into a globally complete and consistent dataset using the laws of atmospheric science. The ERA5 datasets used here are ERA5-Single level^17^ and ERA5-Land^18^. An overview of all ERA5 sub-datasets can be found in the official ERA5 data documentation (https://confluence.ecmwf.int/display/CKB/ERA5%3A+data+documentation, accessed on 27 April 2022). ERA5-Single level provides hourly estimates for various atmospheric and land-surface quantities with a regular grid scheme at various atmosphere levels. ERA5-Land provides near-surface variables over several decades at an enhanced resolution compared to the ERA5-Single level. ERA5-Single level and ERA5-Land datasets can be downloaded through the Climate Data Store portal (https://cds.climate.copernicus.eu/cdsapp#!/home, accessed on 27 April 2022) on a regular latitude/longitude grid of 0.25° × 0.25° and 0.1° × 0.1°, respectively. To get an overview of WE variables selected from ERA5 datasets, Table 4 summarises the WE variables, their sources, descriptions and units.

**Table 4:** WE variables selected from ERA5 datasets.

Dataset	Variable	Description	Unit
ERA5 Land	10 m u-component of wind	Eastward component of the wind at 10 metres altitude	*m*/*s*
	10 m v-component of wind	Northward component of the wind at 10 metres altitude	*m*/*s*
	2 m dewpoint temperature	Temperature to which the air, at 2 metres above the surface of the Earth, would have to be cooled for saturation to occur	*K*
	2 m temperature	Temperature of air at 2 metres above the surface of land, sea or inland waters	*K*
	Total precipitation	Accumulated liquid and frozen water, comprising rain and snow that falls to the Earth’s surface	*m*
	Surface net solar radiation	Amount of solar radiation that reaches a horizontal plane at the surface minus the amount reflected by the Earth’s surface	*J*/*m*^2^
ERA5 Single Level	100 m u-component of wind	Eastward component of the wind at 100 metres altitude	*m/s*
	100 m v-component of wind	Northward component of the wind at 100 metres altitude	*m/s*
	Boundary layer height	Depth of air next to the Earth’s surface that is most affected by the resistance to the transfer of momentum, heat or moisture across the surface	*m*
	Surface pressure	Pressure (force per unit area) of the atmosphere at the surface of land	*Pa*
	Precipitation type	Type of precipitation on the Earth’s surface. Values of precipitation type are: no precipitation (0), rain (1), freezing rain (3), snow (5), wet snow (6), mixture of rain and snow (7), ice pellets (8)	*Categorical*

Relative humidity is useful for studying air quality, so we complete the weather variables by calculating relative humidity. Using the temperature (*T*) and dew point temperature (*T*_*dew*_), we compute the relative humidity (*RH*) using the August-Roche-Magnus approximation formula^19^:

1 $$RH=100\times \exp \left(\frac{17.625\times {T}_{dew}}{243.04+{T}_{dew}}-\frac{17.625\times T}{243.04+T}\right)$$

#### Emissions

The Copernicus Atmosphere Monitoring Service (CAMS) implemented by the ECMWF is one of the most recent global databases covering anthropogenic source emissions. CAMS datasets are compiled emission inventories for many atmospheric compounds developed for the years 2000–2020^20,21^. These inventories are based on a combination of existing datasets and new information, describing anthropogenic emissions from fossil fuel use on land, natural emissions from vegetation, soil and more. The anthropogenic emissions on land are further separated into specific activity sectors (e.g. traffic, agriculture). Pollutant emissions data are provided by the CAMS-anthropogenic emissions dataset (https://permalink.aeris-data.fr/CAMS-GLOB-ANT, accessed on 27 April 2022), which contains monthly global anthropogenic and natural emissions from 36 sources on a regular grid level. The anthropogenic sources are divided into 20 sectors (including agriculture and livestock) with a spatial resolution of 0.1° × 0.1°. Table 5 summarises the emission variables selected, their origins, descriptions and units.

**Table 5:** EM variables selected from the CAMS-anthropogenic emissions dataset.

Variable	Emission origin	Description	Unit
NH_3_	Livestock sector	Emissions of NH3 originating from the livestock sector for manure management	*kg*/(*m*^2^ s)
	Agriculture soils	Emissions of NH3 originating from agriculture soils	*kg*/(*m*^2^ s)
	Waste burning	Emissions of NH3 originating from agriculture waste burning	*kg*/(*m*^2^ s)
	Total	Total emissions of NH3 across all sectors	*kg*/(*m*^2^ s)
NO_*x*_	Road transportation	Emissions of NOx from on road transportation	*kg*/(*m*^2^ s)
	Total	Emissions of NOx across all sectors	*kg*/(*m*^2^ s)
SO_2_	Total	Emissions of SO2 across all sectors	*kg*/(*m*^2^ s)

#### Livestock

The Agrimonia dataset focuses on data on pigs and bovines because of their impact on the Lombardy air quality. In fact, from the Lombardy emission inventory for 2019 (INEMAR^4^), we see that about 87% of the annual ammonia emissions in the study region is estimated to come from slurry and manure management of these species. The related emission pattern has a remarkable spatial variability in the study region and is similar to 2014^22^.

**Fig. 3:**
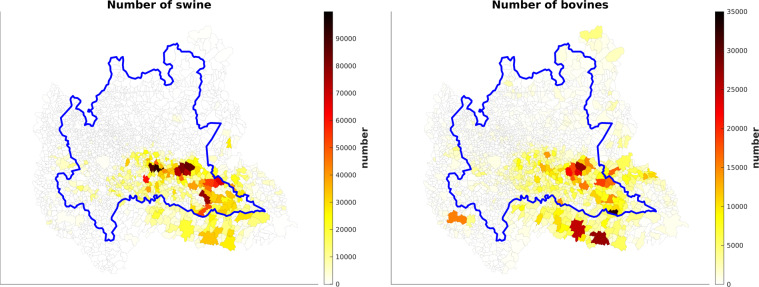
Number of swine (left) and bovines (right) in the augmented Lombardy region and neighbouring area, aggregated at the municipal level on 31 December 2021.

The number of livestock per municipality is obtained by the Italian National Data Bank of the Zootechnical Registry (BDN)^23^, deriving from the livestock census in Italy. The BDN dataset is managed by the Directorate General for Animal Health and Veterinary Medicines (Direzione Generale della Sanità Animale e dei Farmaci Veterinari, https://www.salute.gov.it/portale/temi/p2_5.jsp?area=sanitaAnimale&menu=tracciabilita, accessed on 15 February 2022), of the Italian Ministry of Health and represents the official source of data on livestock, both for the control authorities and users. The dataset is accessible through the “statistics” section of the BDN portal (https://www.vetinfo.it/j6_statistiche/index.html#/, accessed on 15 February 2022). The BDN data are updated every six months and aggregated at the municipality level. Figure 3 shows the number of swine and bovines in the augmented Lombardy region, which is particularly high in the Southeastern areas.

#### Land

The land cover, land and soil use are potentially important factors to assess the agriculture impact on air quality^1,24^. For the land cover, we consider high and low vegetation indexes obtained by the ERA5-Land^18^ dataset already introduced in the Weather Section. For land use, we consider the Corine Land Cover (CLC)^25^ dataset handled by the Copernicus Land Monitoring Service (CLMS). It is available for 2018 and consists of an inventory of land use in 44 classes within a minimum mapping unit of 25 hectares. The classes are organised in the hierarchical 3-level CLC nomenclature^26^ based on the classification of satellite images. The CLC dataset has been downloaded from the CLMS portal (https://land.copernicus.eu/pan-european/corine-land-cover, accessed on 27 April 2022). The information on soil use and cultivation type is obtained by the Agriculture Information System of the Lombardy Region (SIARL)^27^, which provides the classification of agricultural soil use in 21 classes at yearly frequency, and is available until 2019.

Table 6 summarises the above land cover, land and soil use variables. In the Agrimonia repository^6^, the detailed labels for CLC and SIARL categorical variables are available in the files named ‘Metadata_LA_CORINE_labels.csv’ and ‘Metadata_LA_SIARL_labels.csv’ respectively.

**Table 6:** LA variables selected from the ERA5, CLC and SIARL datasets.

Dataset	Variable	Description	Unit
ERA5-Land (land cover)	Low vegetation index	One-half of the total green leaf area per unit horizontal ground surface area for low vegetation type	*m*^2^/*m*^2^
	High vegetation index	One-half of the total green leaf area per unit horizontal ground surface area for high vegetation type	*m*^2^/*m*^2^
CLC (land use)	Third-level of land use	Classification of land use with 44 classes	*Categorical*
SIARL (soil use)	Soil cultivation type	Classification of soil use with 21 classes	*Categorical*

### Data harmonisation and processing

Since the previous section discussed the input data’s different spatial and temporal resolutions, we introduce here the methods used to harmonise the data before merging them into the Agrimonia dataset. We also consider missing data imputation and some variable transformations. In the metadata file named ‘Metadata_Agrimonia.csv’, provided along with the Agrimonia dataset^6^, columns 9–14 summarise the original spatial and temporal resolutions and the transformations converting the variables to the same spatial and temporal resolution, i.e., daily quantities at the station level.

#### Air quality

The AQ data, from ARPA and EEA networks, have both short and prolonged periods with stations turned off for maintenance, instrument calibration or other reasons. In some cases, measurements are taken at irregular intervals, or sampling policies change. In fact, Table 2 shows that, daily, bi-hourly and hourly AQ measurements are present in the network. We have detected the frequency of each time series automatically while considering the presence of various kinds of missing data. To do this, we computed the distribution of the corresponding time gaps between the measures and used the resulting mode to identify the temporal resolution.

Since the presence of several consecutive missing values in a day may introduce a bias in the daily average, we implemented the missing imputation at the hourly/bi-hourly frequency given by the following algorithm. First, measurements not validated by the environmental agencies and negative values are considered as missing values (‘NaN’). For each time series, missing values are then imputed using a state-space model^28^ and the relative Kalman smoother^29^ providing an estimate of the missing data and their uncertainty. Next, hourly and bi-hourly time series are averaged over each day. Days with a gap larger than six hours are set to missing. The Kalman smoother uncertainty associated with the hourly estimate is propagated to the daily average, thus providing daily uncertainties due to the missing data imputation. Details on this approach are discussed in the Technical validation Section. The resulting imputation uncertainty is reported in the metadata file ‘Metadata_AQ_imputation_uncertainty.csv’ provided along with the Agrimonia dataset^6^. Table 7 shows the missing data percentage by year for the different pollutants remaining after harmonisation.

**Table 7:** Missing data of AQ variables in the Agrimonia dataset. All AQ variables included in the Agrimonia dataset are harmonised to the daily time resolution through the Kalman smoother and the daily mean. More details on the transformation process can be found in the metadata file named ‘Metadata_Agrimonia.csv’ available with Agrimonia dataset^6^. The columns starting with ‘%’ show the percentage of missing data after harmonisation for each pollutant grouped by year. Note that these percentages refer to the sensor numbers listed in Table 2 only.

Variable name	Description	% 2016	% 2017	% 2018	% 2019	% 2020	% 2021
AQ_pm_10	Particulate matter with an aerodynamic diameter of less than 10 µm	6	6.3	4.7	5.7	4.4	11.6
AQ_pm_2.5	Particulate matter with an aerodynamic diameter of less than 2.5 µm	15	12.7	10.6	10.4	9.9	31
AQ_co	Carbon monoxide	8.3	8.4	6.2	6.8	9.7	13.8
AQ_nh3	Ammonia	33.8	22.7	29.7	28.7	16.3	18.5
AQ_nox	Several oxides of nitrogen	23.9	24.7	6.2	5.4	16.1	23
AQ_no2	Nitrogen dioxide	8.6	15.2	14.1	13	12	14
AQ_so2	Sulphur dioxide	9.6	7.6	5.6	5.5	3.2	7.2

#### Weather

This section describes in detail the harmonisation process for the WE variables (see Table 8). The data about WE come from ERA5 datasets and are given by hourly reanalysis estimates in a regular grid format. It should be noted that the ERA5 value refers to the grid cell average, while the coordinates refer to the centre of the cell. Because the data stem from a model, there is no problem with missing and negative values. Some variables need to be preprocessed to be more informative. We summarise the two preprocessing steps for the original variables, as follows: the wind speed is calculated as the Euclidean norm of the wind vector with *u-* and *v-* components; the wind direction is discretised using the classical 8-wind rose: North (N), North-east (NE), East (E), South-east (SE), South (S), South-west (SW), West (W), North-west (NW); the temperature is converted from Kelvin to Celsius degrees.

**Table 8:** WE variables included in the Agrimonia dataset. More detail on the transformation process can be found in the metadata file named ‘Metadata_Agrimonia.csv’ available with Agrimonia dataset^6^.

Variable name	Description	Temporal aggregation function	Units
WE_temp_2m	Temperature of air at 2 m above the surface of land, sea or inland water	Daily mean	°*C*
WE_wind_speed_10_mean	Mean intensity of the wind speed at a height of 10 m above the surface of the Earth	Daily mean	*m/s*
WE_wind_speed_10_max	Max intensity of the wind speed at a height of 10 m above the surface of the Earth	Daily max	*m/s*
WE_mode_wind_direction_10m	Direction of the wind intensity at a height of 10 m above the surface of the Earth	Daily mode	*Categorical*
WE_tot_precipitation	The accumulated liquid and frozen water, comprising rain and snow, that falls to the Earth’s surface	Daily sum	*m*
WE_precipitation_t	The type of precipitation on the Earth’s surface, at the specified time. Values of precipitation type are: no precipitation (0), rain (1), freezing rain (3), snow (5), wet snow (6), mixture of rain and snow (7), ice pellets (8)	Daily mode	*Categorical*
WE_surface_pressure	The pressure (force per unit area) of the atmosphere at the surface of land, sea and inland water	Daily mean	*Pa*
WE_solar_radiation	Amount of solar radiation that reaches a horizontal plane at the surface minus the amount reflected by the Earth’s surface	Daily max	*J/m*^2^
WE_wind_speed_100_mean	Mean intensity of the wind speed at a height of 10 m above the surface of the Earth	Daily mean	*m/s*
WE_wind_speed_100_max	Max intensity of the wind speed at a height of 10 m above the surface of the Earth	Daily max	*m/s*
WE_mode_wind_direction_100m	Direction of the wind intensity at a height of 100 m above the surface of the Earth	Daily mode	*Categorical*
WE_blh_layer_max	The maximum depth of air next to the Earth’s surface that is the most affected by the resistance to the transfer of momentum, heat or moisture across the surface	Daily max	*m*
WE_blh_layer_min	The minimum depth of air next to the Earth’s surface that is the most affected by the resistance to the transfer of momentum, heat or moisture across the surface	Daily min	*m*
WE_rh_min	Maximum amount of water vapour present in air expressed as a percentage of the amount needed for saturation at the same temperature	Daily min	%
WE_rh_mean	Mean amount of water vapour present in air expressed as a percentage of the amount needed for saturation at the same temperature	Daily mean	%
WE_rh_max	Minimum amount of water vapour present in air expressed as a percentage of the amount needed for saturation at the same temperature	Daily max	%

**Fig. 4:**
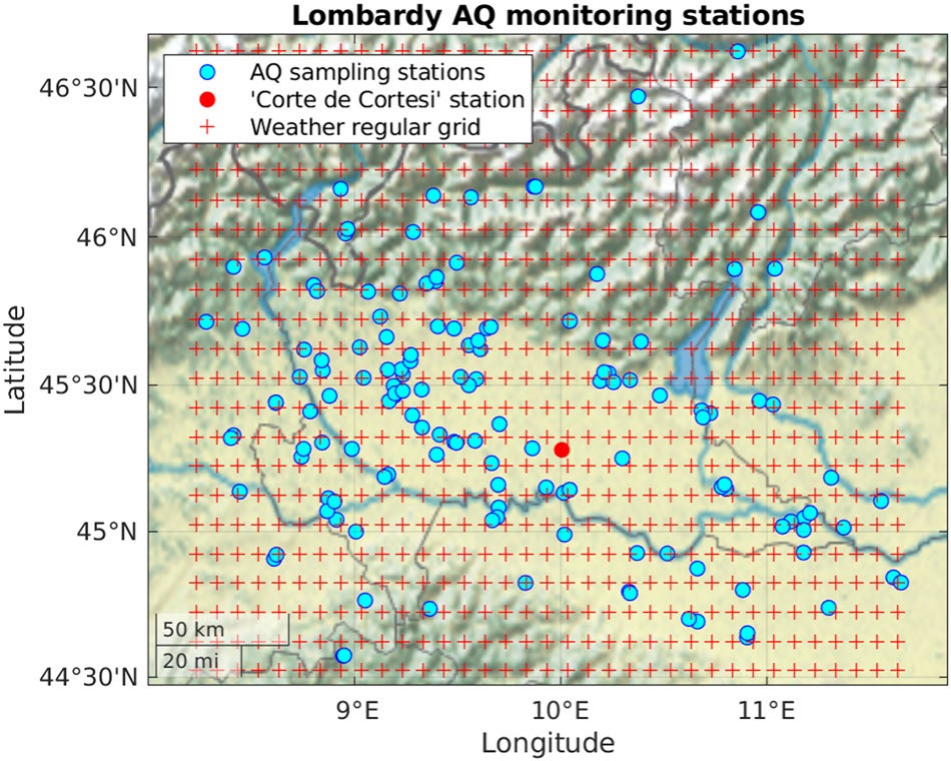
The irregularly located AQ monitoring stations (cyan circles) and the weather grid centres (red ‘+’ symbols). Station named ‘Corte de Cortesi’ is marked as a red circle.).

**Fig. 5:**
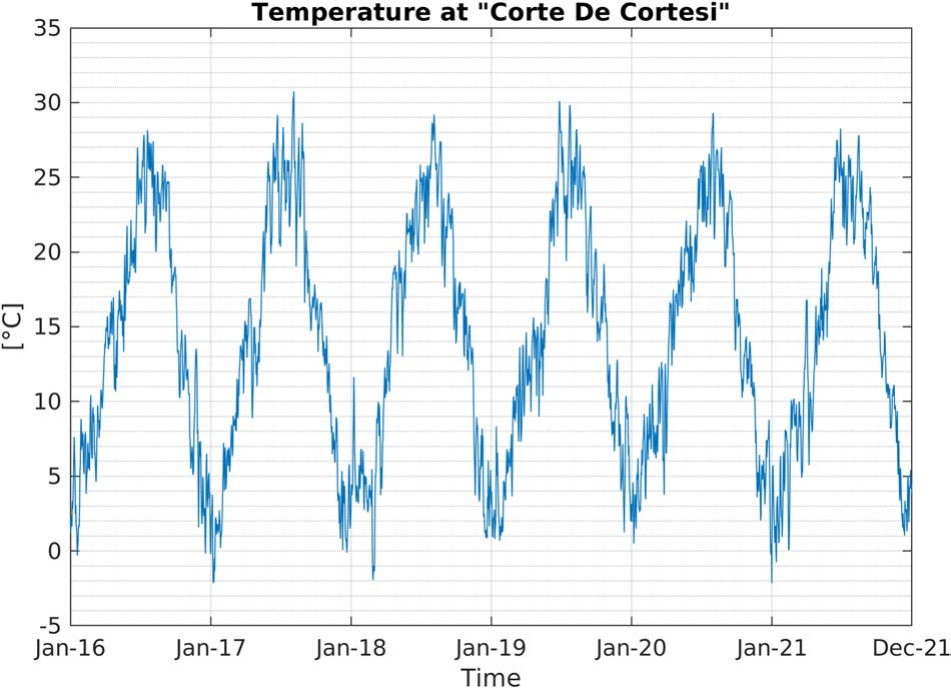
Daily 2 m temperature time series (WE_temp_2m) from 2016 to 2021 for the monitoring station named ‘Corte De Cortesi’ (see red point in Fig. 4).

The transformation of weather variables to create daily time series is composed of two different stages. The first one is to create an hourly weather time series related to each AQ monitoring station, while the second consists of computing daily time series from hourly time series. The first step is necessary because the AQ station is misaligned concerning weather data, as shown in Fig. 4. To associate weather time series to each AQ station, we use the *inverse distance weighted* (IDW) interpolation algorithm^30^. The IDW algorithm is based on the Euclidean distance between the localisation of the stations and grid cells’ centres. For each station, we consider the four nearest grid cells. The IDW power parameter, which controls the weight of the cell values on the interpolated values based on their distance from the localisation of the station, is set to one. After the hourly time series is created using the IDW approach for each station, we convert the temporal resolution from hourly to daily using different ensemble functions according to the variable type^31^. Table 8 lists the weather variables in the Agrimonia dataset while Fig. 5 shows an example of time series obtained using the IDW approach.

#### Emissions

This section describes the harmonisation process for the EM variables summarised in Table 9. The data about EM are from the CAMS datasets with a monthly temporal resolution and on a regular grid. As done for the WE variables, we performed a two-step transformation process to create daily emission time series. In the first step, we use the same IDW approach described for WE variables to compute emission values related to each monitoring station with monthly resolution. In the second step, we use spline interpolation techniques to convert the series to the same daily temporal resolution. To avoid oscillations, overshoots, edge effects and negative values, we use *piecewise cubic Hermite interpolating polynomials* (PCHIP)^32^. As discussed in more detail in the Technical validation Section, this method interpolates the data smoothly, while retaining the data’s shape and monotonicity.

**Table 9:** Pollutant EM variables [*mg/m*^2^] present in the Agrimonia dataset with daily temporal resolution. For each EM variable we use IDW function as spatial transformation and PCHIP interpolation function to transform form monthly to daily temporal resolution. More detail on the transformation process can be found in the metadata file named ‘Metadata_Agrimonia.csv’ available with Agrimonia dataset^6^.

Variable name	Description
EM_nh3_livestock_mm	Emissions of NH_3_ originating from the livestock sector for the manure management
EM_nh3_agr_soils	Emissions of NH_3_ originating from agriculture soils
EM_nh3_agr_waste_burn	Emissions of NH_3_ originating from the burning of agriculture waste
EM_nh3_sum	Total emissions of NH_3_ across all sectors (anthropogenic)
EM_nox_traffic	Emissions of NO_*x*_ from the on-road transportation
EM_nox_sum	Emissions of NO_*x*_ across all sectors (anthropogenic)
EM_no2_sum	Total emissions of SO_2_ across all sectors (anthropogenic)

#### Livestock

**Fig. 6:**
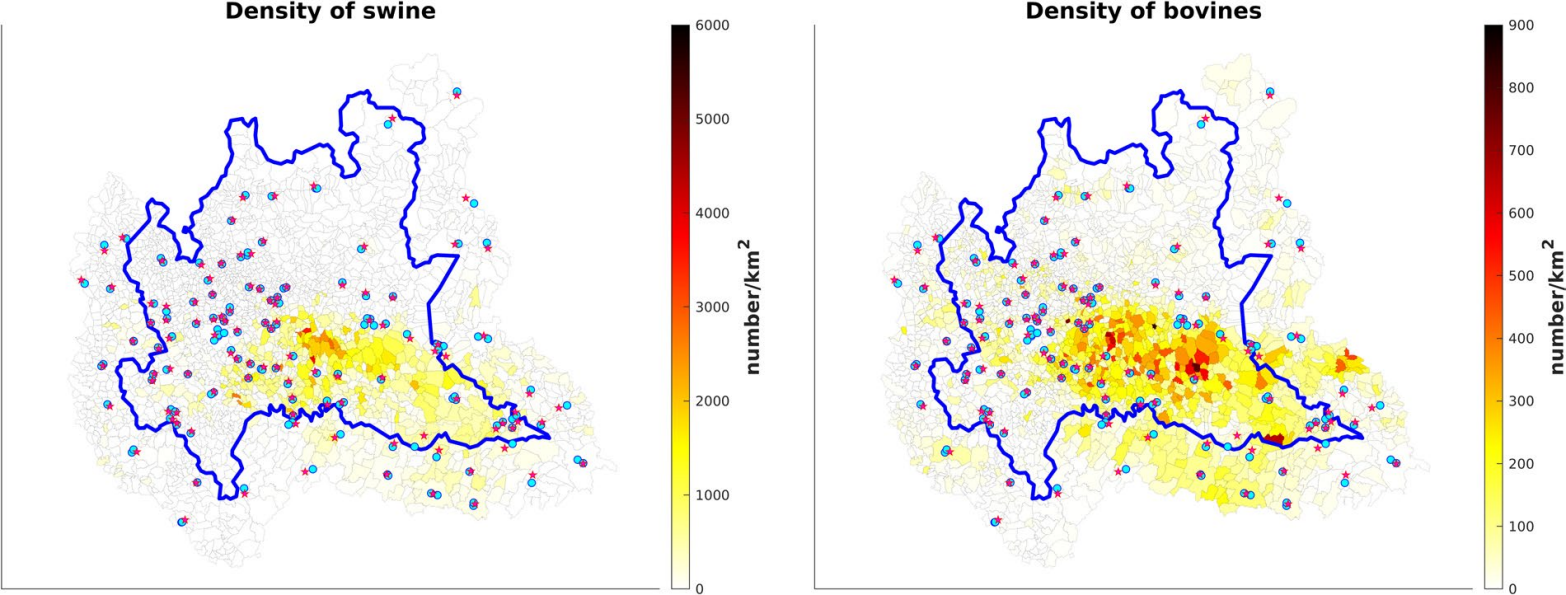
Swine (left) and bovines (right) density over the augmented Lombardy region on 31 December 2021. The AQ stations (cyan circle) are spread randomly over the studied area. Each station is associated with the the municipality centroid to which it belongs (red start). Based on this, stations in the same municipality share the same municipality centroid so they have the same livestock time series.

In this section, we describe the harmonisation process for the LI variables summarised in Table 10. The data related to the livestock sector are retrieved from the BDN dataset, which provides the number of bovines and swine aggregated at the municipality level. The BDN dataset is updated every six months, in June and in December. As a result, for each municipality, a time series of 12 values are available. Each AQ station is associated with the time series of the municipality to which it belongs, see Fig. 6 for swine and bovines. Due to the particular municipality shape, the station named ‘Vallelaghi_T1191A’ is within a municipality whose centroid is outside of the considered augmented domain, therefore the value of the closest municipality in the area considered is taken.

**Table 10:** LI variables in the Agrimonia dataset with daily temporal resolution. Information on the number of swine and bovines is expressed as a density with respect to the municipal area: *number/km*^2^. More detail on the transformation process can be found in the metadata file named ‘Metadata_Agrimonia.csv’ available with Agrimonia dataset^6^.

Variable name	Description
LI_pigs	Municipal density of swine related to AQ stations
LI_bovine	Municipal density of bovines related to AQ stations

As done for the CAMS data, the PCHIP interpolation is used to increase the temporal resolution from biannual to daily (for more details, see the Technical validation Section). Once the interpolation function has been chosen, it is possible to evaluate it over the entire time horizon, particularly for all daily instants. To reduce edge effects, we use the value for December 31, 2015, as the first starting value for the time series. Subsequently, the municipal animal density is calculated by dividing the animal count by the area of the station municipality (expressed in *km*^2^). In this way, we obtain the daily time series of swine and bovines density for each monitoring station. See Table 10 for a summary of the livestock variables in the Agrimonia dataset.

#### Land

This section describes in detail the harmonisation process for the LA variables, which are summarised in Table 11. The data related to land cover and land and soil use are given by the ERA5-Land, CLC and SIARL datasets, respectively, describing the land and soil over time. Considering that high and low vegetation indices from ERA5-Land have a daily resolution over a spatial regular grid, we use the same IDW approach described for WE variables to create the daily time series associated with each AQ station. Information about land use is relatively stable over time. For the CLC dataset, we take the 2018 data and keep them constant for the period from 2016 to 2021. For the SIARL dataset, the values are annual until 2019 (for more detail on land use and land cover, see the Technical validation Section). The CLC provides categorical data in polygons while SIARL does so on a regular grid. In both cases, each AQ station is associated with the polygon or the cell to which it belongs. In the metadata files provided along with the Agrimonia dataset^6^, the class labels for CLC and SIARL datasets are available in the files named ‘Metadata_LA_CORINE_labels.csv’ and ‘Metadata_LA_SIARL_labels.csv’, respectively. In this way, we obtain daily piecewise constant functions for land cover and soil use associated with each AQ station.

**Table 11:** LA variables in Agrimonia dataset with daily time resolution. More detail on the transformation process and label for categorical variables can be found in the metadata files named ‘Metadata_Agrimonia.csv’, ‘Metadata_LA_CORINE_labels.csv’ and ‘Metadata_LA_SIARL_labels.csv’, respectively, provided with Agrimonia dataset^6^.

Variable name	Description	Spatial transformation	Temporal transformation	Unit
LA_hvi	One-half of the total green leaf area per unit horizontal ground surface area for high vegetation type	IDW	None	*m*^2^/*m*^2^
LA_lvi	One-half of the total green leaf area per unit horizontal ground surface area for low vegetation type	IDW	None	*m*^2^/*m*^2^
LA_land_use	CORINE Land Cover - Land use across 44 sectors	None	None	*Categorical*
LA_soil_use	SIARL Lombardy - Lombardy soil use across 21 sectors	None	None	*Categorical*

## Data Records

The output dataset has been built by joining the daily time series related to the air quality (AQ), weather (WE), emission (EM), livestock (LI) and land (LA) variables discussed in the previous sections and referred to the same AQ monitoring station for the Lombardy region augmented by the 0.3° buffer, depicted in Fig. 1. The dataset (version 2.0.1) and the metadata files are available on the Zenodo repository^6^ as follows:

- *Agrimonia_Dataset_v_2_0_1.csv*: this is the Agrimonia output dataset joining the daily time series, at station locations, related to the AQ (see Table 7), WE (see Table 8), EM (see Table 9), LI (see Table 10) and LA (see Table 11) variables. In order to simplify the access to variables in the Agrimonia dataset, the variable name starts with the dimension of the variable, e.g., the name of the variables related to the AQ dimension starts with ‘AQ_’. Missing data are denoted by the ‘NaN’ value. The dataset has *S* = 141 monitoring stations and *T* = 2192 days between 1st January 2016 and 31st December 2021, totalling *S* × *T* = 309072 rows, with the unique identifier given by the pair station code and date in “YYYY-MM-DD” format. It contains 41 columns, including the following block: row header (station code, latitude and longitude, date and altitude), AQ (7 columns), WE (16 columns), EM (7 columns), LI (2 columns) and LA (4 columns). This file is made available also in the *.mat* and *.Rdata* format for MATLAB and R software, respectively.
- *Metadata_Agrimonia.csv*: this is the main Agrimonia metadata file and provides further information for the sources used, variables imported, transformations applied and Agrimonia variables.
- *Metadata_monitoring_network_registry_v_2_0_1.csv*: it contains details about the AQ monitoring stations, including station type, NUTS3 code, environment type, altitude, monitored pollutants and others. Each row represents a single sensor.
- *Metadata_AQ_imputation_uncertainty.csv*: it contains the estimate of the daily uncertainty due to missing data imputation for the AQ time series. In particular, for each AQ variable, days without missing hours have zero uncertainty, days with one or more imputed hours have a number resulting from the propagation of the uncertainty in the daily averaging, and days with a ‘NaN’ in the concentrations have a ‘NaN’ also in the uncertainty.
- *Metadata_LA_CORINE_labels.csv*: it contains labels and descriptions associated with the CLC land variables (column CORINE code).
- *Metadata_LA_SIARL_labels.csv*: it contains labels and descriptions associated with the SIARL land variables (column SIARL code).

### Data availability, abundance and standards

The Agrimonia dataset origins from a multi-pollutant monitoring network, where not each measurement station was equipped with all sensors. In addition to this heterogeneity, maintenance of the AQ monitoring network and temporal and/or geographic constraints of the CAMS, BDN and SIARL datasets lead to a varying data availability. For convenience, the dataset is published in tabular format with size 309072 rows × 41 columns and all variables are included for each station and time point. If a sensor/measurement was not available, the values are replaced with (pseudo-)‘NaN’ values. Even though this tabular format increases the required storage capacity, modern data analysis software can efficiently handle the related large number of ‘NaN’ values.

In particular, for AQ, the number of stations per pollutant is reported in Table 2 with an average of 73, about one-half of the 141 stations. The number of pseudo-random missing data per year of active sensors is reported in Table 7. As a result, about 54% of the AQ cells are set to ‘NaN’ in the Agrimonia dataset. For EM, data are available only up to 2020. For LI, the three stations in Switzerland have missing data for bovines and swine because the BDN census data is available only in Italy. Similarly, for LA soil use, SIARL data is available only in Lombardy up to 2019. Hence the geographic buffer of 48 stations and the last two years are set at ‘NaN’ in our dataset. Considering the above-mentioned aims of the AgrImOnIA project, the missing values outside Lombardy for LA and LI predictors are not a concern due to the focus on the region and the auxiliary role of the buffer. Instead, the lack of SIARL data after 2019 limits the possibility of using the cultivation classification for the entire time series.

The Agrimonia dataset is consistent with the following reference systems: World Geodetic System 1984 (WGS84)^33^ for georeferentiation, Coordinated Universal Time (UTC) for time referentiation and the International System of Units (SI) for the metric system, except for the temperature expressed in Celsius degrees (°*C*). The values in the dataset are represented in scientific notation with four significant digits here, considered sufficient for statistical modelling purposes. The coordinates (latitude, longitude) have a fixed point representation with nine significant digits to identify the stations’ locations correctly.

## Technical Validation

### Validation of AQ variables

The last column of Table 2 shows that the AQ network, intended as a multipollutant monitoring network, is unbalanced as each sensor is settled according to a pollutant-specific risk and exposure assessment, following EU regulations. As a result, the various stations often have different sensors. Considering CO sensors, we do not see a big problem for environmental understanding, as CO is well below health standards in Lombardy and is not strongly related to livestock. Unfortunately, NH_3_ sensors are very few.

Hence, due to the table structure, the Agrimonia dataset has “systematic” missing values for a specific pollutant in all those stations which do not monitor that quantity. In addition, most sensors are subject to maintenance and other issues resulting in quasi-random missing values. NH_3_ sensors are not required by EU regulations and are somewhat experimental, as explained above. This results in long non-operating periods.

Providing a complete dataset with all (systematic ad quasi-random) missing data imputed by some estimation method is an interesting objective. It may be pursued using three approaches.

- A suitable approach is based on multivariate statistical models applied to the unbalanced network and some additional auxiliary variables, see e.g.^34^.
- Mathematical and numerical techniques may be used to simulate the physical and chemical processes that affect air pollutants as they disperse and react in the atmosphere. For example, see the Models-3/Community Multiscale Air Quality (CMAQ; http://www.epa.gov/asmdnerl/CMAQ) model.
- A mixture of numerical and statistical models may be used to leverage over the previous two; see e.g.^35^. Indeed, AgrImOnIA project is involved in this task for PM_10_ and PM_2.5_, but this is out of the scope of the current paper and dataset.

The missing values imputation for the hourly and bi-hourly time series is performed using the State-Space Model (SSM)^28^ and the relative Kalman smoother^29^. For any hour *t∈* {1, ⋯, 24*T*}, where *T* is the number of days as before, let *x*_*t*_ be the scalar state describing the dynamics of the underlying AQ “true” concentrations and let *y*_*t*_ be the scalar observation of the observed hourly AQ series. Moreover, let *u*_*t*_ and *ε*_*t*_ be Gaussian white noises with unit-variance representing the innovation and measurement error, respectively, with *u*_*t*_ and *ε*_*t*_ uncorrelated. The SSM here used for missing imputations is defined by:

2 $$\left\{\begin{array}{ccc}{x}_{t} & = & A{x}_{t-1}+B{u}_{t}\\ {y}_{t} & = & {x}_{t}+{\varepsilon }_{t}\end{array}\right.$$

where the *A* and *B* parameters describe the dynamics and the additive error structure on the state *x*_*t*_, respectively. Both *A* and *B* are estimated for each hourly time series using numerical optimisation of the likelihood function with initial values set to one. Assuming the hourly state errors ${x}_{t}-{\widehat{x}}_{t}$ uncorrelated, we propagate the imputation uncertainty given by the smoother, through the mean of the generic day (*d*) as ${\sigma }_{d}=\sqrt{1/2{4}^{2}{\sum }_{t\in d}Var({x}_{t}\,| \,{y}_{1},...,{y}_{24T})}$ where $Var({x}_{t}\,| \,{y}_{1},...,{y}_{24T})$ is the variance of the smoothed states *x*_*t*_ for the hour *t ∈* {1, ⋯, 24*T*}.

**Fig. 7:**
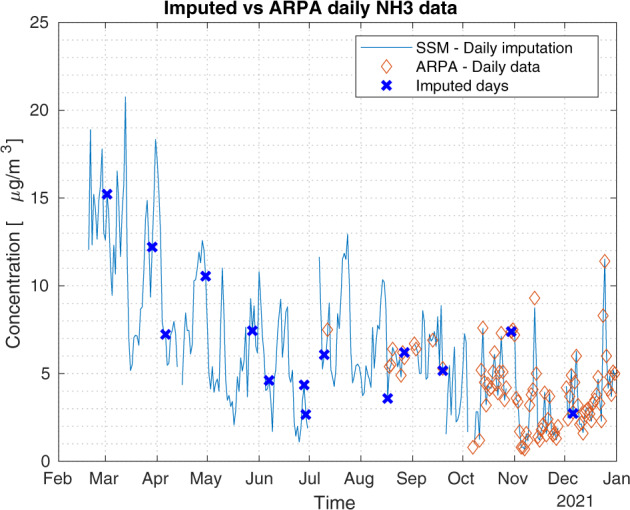
Hourly NH_3_ data. Impact of Kalman smoother on daily data for the monitoring station named ‘Bergamo Via Meucci’. Daily time series obtained with our method (blue line) with highlighted imputed days (blue crosses) and daily raw data (orange diamonds), RMSE = 0.1710.

**Fig. 8:**
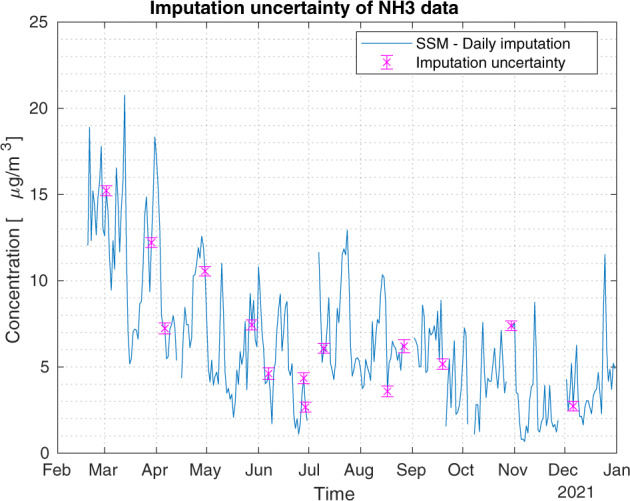
Hourly NH_3_ data. Impact of Kalman smoother on daily data for the monitoring station named ‘Bergamo Via Meucci’. Imputed daily data (blue line) with associated imputed uncertainty (±2*σ*_*d*_, magenta bar).

A validation experiment concerns NH_3_ at the station called ‘Bergamo Via Meucci’, where ARPA Lombardy provides both hourly and daily time series. The daily time series is validated by ARPA but has a shorter coverage than the hourly time series. Indeed, the latter is not validated by ARPA as discussed in the Method Section and has several missing values; see Fig. 7. The idea is to use the longer hourly time series to reconstruct the daily time series by our proposed method. So, we verify the performance of the missing imputation process by comparing the daily data obtained through the Kalman smoother and the daily data provided by the agency. Figure 7 shows the two daily time series: the blue line depicts our method while the orange diamonds are the ARPA Lombardy daily data. From Fig. 7, we see that the ARPA daily data and the daily mean of the hourly data may not be exactly the same in some cases, also for those days without missing values. This is due to the validation process performed by the ARPA on the daily data. Figure 8 shows the imputed uncertainty of daily average concentrations computed from imputed hourly time series. It can be observed that the time series obtained by our missing imputation method is very close to the daily one, with the Root Mean Square Error (RMSE) equal to 0.1710.

**Fig. 9:**
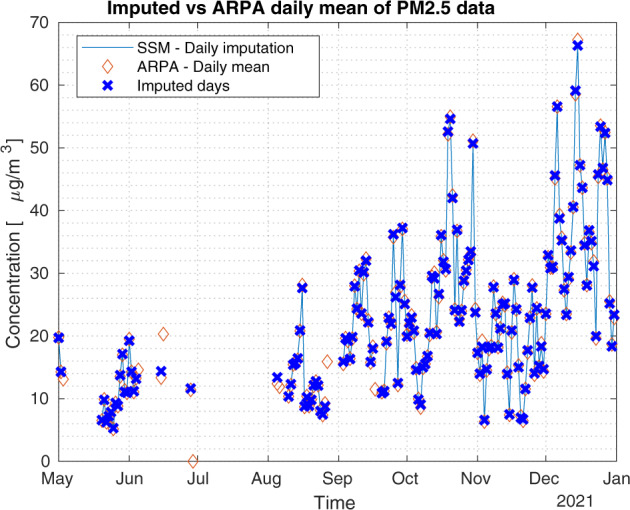
Bi-hourly PM_2.5_ data. Impact of Kalman smoother on daily data for the monitoring station named ‘Parona Via della Miseria’. Daily time series obtained with our method (blue line) with highlighted imputed days (blue crosses) and daily mean (orange diamonds) computed without considering missing values, RMSE = 0.3078.

**Fig. 10:**
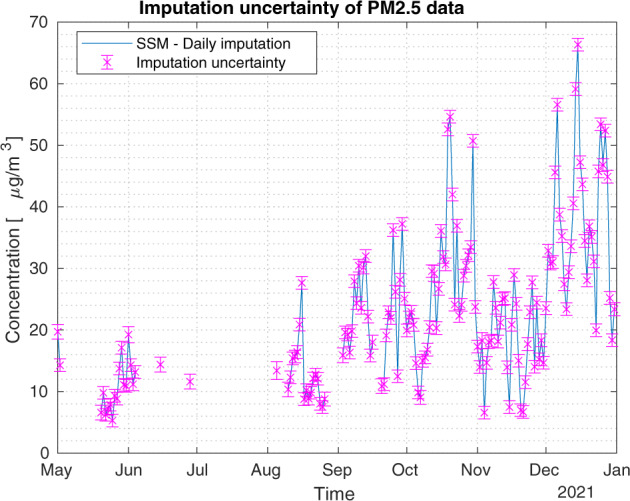
Bi-hourly PM_2.5_ data. Impact of Kalman smoother on daily data for the monitoring station named ‘Parona Via della Miseria’. Imputed daily data (blue line) with associated imputed uncertainty (±2*σ*_*d*_, magenta bar).

Another important experiment concerns the stations that sample PM_2.5_ bi-hourly. In this case, if the sampling frequency is regular during the day, we do not expect bias problems in the associated daily time series, although we still have 12 missing values for each day spread every other hour. This situation occurs, for example, in the station called ‘Parona Via della Miseria’. In fact, the data on PM_2.5_ concentrations are available from May 2021, are validated and have a bi-hourly frequency. Figure 9 shows the daily mean computed with our approach (blue line) and the imputed days (blue crosses) compared to the daily mean (orange diamonds) computed without considering missing values. Instead, Fig. 10 draws the imputed uncertainty of daily average concentrations computed from imputed hourly time series and shows that it is small.

We examined the dataset for the presence of anomalous values that are clearly outliers after the daily time series construction. Table 12 lists the three instances of anomalous values found. These extreme values have been replaced with the ‘NaN’ value. It is to be noted that this process is not to be considered a process of searching and removing outliers which is outside the context of this work.

**Table 12:** Extremely large values for the AQ variables [*μg*/*m*^3^] identified in the dataset. The values are replaced with the ‘NaN’ value.

IDStations	Latitude	Longitude	Time	Pollutant	Value
STA.IT1582A	45.1361	8.4452	2021-04-27	AQ_pm_10	2399
STA.IT2121A	45.6894	8.4584	2021-04-04	AQ_pm_25	2794
STA.IT1751A	44.5740	8.9510	2016-10-10	AQ_so2	152.03

### Validation of WE variables

ARPA meteorological sensors effectively understand local meteorological conditions at the station site. This data has been used in various studies to adjust air quality estimates for meteorology (see e.g.^24^.) The AgrImOnIA project aims at producing maps of the livestock impact on AQ, that is, estimates of air quality and relations where no stations are available. For this reason, we used the ERA5 dataset, which provides average meteorological conditions over each pixel 0.25° × 0.25°. This means that ERA5 is not aimed at reproducing local conditions at the station. Nonetheless, the issue of which meteorological data provides a better predictor for air quality in Lombardy is an open issue and deserves attention for future research.

### Validation of EM and LI variables

Livestock emission variables may be retrieved either from the emission inventory of the Lombardy region known as INEMAR^4^ or from the above-mentioned Copernicus product here named CAMS. We prefer CAMS as it fulfils the aims of the AgrImOnIA project mentioned in the Introduction section, namely generalisability and mappability. In particular, CAMS provides spatially resolved information monthly, whilst INEMAR provides municipally aggregated data yearly. The former is also readily available for the extended Lombardy, which overlaps with other Italian regions and Switzerland. Hence it is easier for inter-region and/or inter-country comparisons.

The original time resolution for EM and LI variables is lower than daily. In particular, EM variables are provided with a monthly temporal resolution, while LI variables are available every six months. Interpolation techniques are required for daily estimates.

We should consider that EM variables depend on manure management and spreading calendar, which are not continuos over time but concentrated on certain days for each farm. Similarly, the basic quantities for LI are livestock counts changing according to population dynamics driven by animal births, deaths, sales and purchases. Especially the latter two are often concentrated in certain time moments. Hence, the following interpolation splines must be interpreted as smooth approximations of an underlying process with irregular steps.

**Fig. 11:**
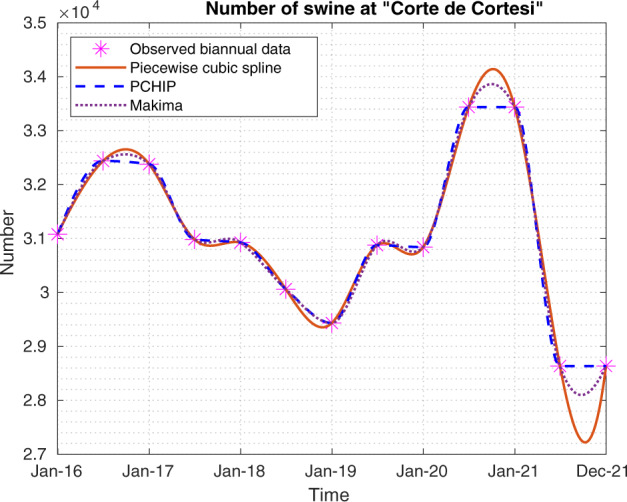
Piecewise cubic spline, PCHIP and Makima interpolation methods applied to swine time series for the monitoring station named ‘Corte de Cortesi’.

To avoid oscillations, overshoots, edge effects and negative values, we use PCHIP^32,36^ interpolation. This method interpolates the data using a piecewise cubic polynomial while retaining the shape and monotonicity of the original data. Since the points to be interpolated are all positive (e.g. the number of bovines), negative values that could occur with classic splines are avoided. For example, Fig. 11 shows the classic piecewise cubic spline, PCHIP and modified Akima piecewise cubic Hermite interpolation (Makima)^37^, fitted on data from the ‘Corte de Cortesi’ station.

### Validation of LA variables

Data related to land cover and land and soil use are taken from ERA5-Land, CLC and SIARL datasets, respectively. The main considerations concern land use and soil use.

Land use classifies the territory from the urbanisation and/or nature point of view (urban, industrial, road, agricultural, forest, marine and other). Since CLC data are available only for 2018 in the study period (2016–2021), we assume the land use to be constant in this work. This seems a minor approximation given that the overall area used by various sectors (such as agriculture and infrastructure) changes slowly over time. Also, this assumption is consistent with the fact that the station type of Table 3 is constant over time for each station.

As an alternative to CLC land use, some authors^38^ use the station type of Table 3. Unfortunately, using station type as an AQ predictor does not allow mapping and/or geostatistical interpolation far away from station locations. Moreover, one could use the ARPA zoning for the Lombardy region^24^. We notice that CLC is defined on a fine grid, which is needed to accomplish the spatially resolved mapping objective of the AgrImOnIA project. Also, the former is better for extended Lombardy and other intercomparisons such as inter-region and/or inter-country comparisons.

**Fig. 12:**
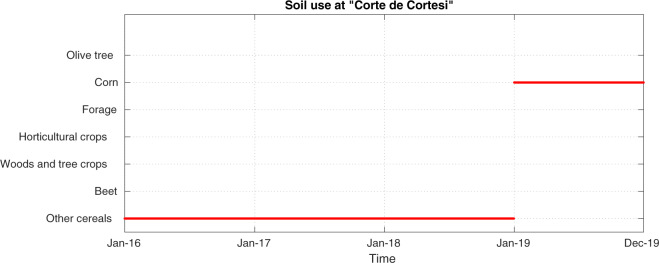
Piecewise constant function for soil use provided by SIARL dataset for the station named ‘Corte de Cortesi’. Note that the SIARL dataset covers data up to 2019 only. The class labels for the SIARL dataset are available in the file named ‘Metadata_LA_SIARL_labels.csv’ available with the Agrimonia dataset^6^.

Soil use classifies the land from the agricultural production point of view (cultivation type). It is expected to change over time as the cultivation type is often changed and/or rotated for greater yield. As an example, Fig. 12 shows the soil use change in 2019 provided by the SIARL dataset for the station named ‘Corte dei Cortesi’.

## Usage Notes

This paper presents an open access spatiotemporal dataset, named Agrimonia dataset^6^, which provides the user with a dataset about ammonia (NH_3_) emissions, agricultural information, air pollution and meteorology in a common spatiotemporal resolution. The dataset is ready to be used ‘as is’ by those using air quality data for research. The Agrimonia dataset and metadata can be accessed through Zenodo^6^ with 10.5281/zenodo.6620529. In the same repository, metadata and supplementary materials are provided to better understand the dataset. As previously mentioned, this dataset was initially compiled for the *AgrImOnIA project* and will be updated when new variables will be available.

## Data Availability

To replicate our work, downloadable helper functions can be used to filter and merge data from different sources. The code used to extract and process all the datasets are developed using Matlab and R. The codes are available at https://github.com/Agrimonia-project/Agrimonia_Data.git, with the user instructions included in the respective ‘README.md’ files.
